# Racial and Socioeconomic Disparities in Complex Abdominal Wall Reconstruction Referrals

**DOI:** 10.3389/jaws.2024.12946

**Published:** 2024-05-30

**Authors:** Alexis M. Holland, Brittany S. Mead, William R. Lorenz, Gregory T. Scarola, Vedra A. Augenstein

**Affiliations:** Division of Gastrointestinal and Minimally Invasive Surgery, Department of Surgery, Carolinas Medical Center, Charlotte, NC, United States

**Keywords:** racial disparity, socioeconomic inequalities, ventral hernia repair, tertiary hospital, abdominal wall reconstruction

## Abstract

**Background:** Health disparities are pervasive in surgical care. Particularly racial and socioeconomic inequalities have been demonstrated in emergency general surgery outcomes, but less so in elective abdominal wall reconstruction (AWR). The goal of this study was to evaluate the disparities in referrals to a tertiary hernia center.

**Methods:** A prospectively maintained hernia database was queried for patients who underwent open ventral hernia (OVHR) or minimally invasive surgical (MISR) repair from 2011 to 2022 with complete insurance and address information. Patients were divided by home address into in-state (IS) and out-of-state (OOS) referrals as well as by operative technique. Demographic data and outcomes were compared. Standard and inferential statistical analyses were performed.

**Results:** Of 554 patients, most were IS (59.0%); 334 underwent OVHR, and 220 underwent MISR. IS patients were more likely to undergo MISR (OVHR: 45.6% vs. 81.5%, laparoscopic: 38.2% vs. 14.1%, robotic: 16.2% vs. 4.4%; *p* < 0.001) when compared to OOS referrals. Of OVHR patients, 44.6% were IS and 55.4% were OOS. Patients’ average age and BMI, sex, ASA score, and insurance payer were similar between IS and OOS groups. IS patients were more often Black (White: 77.9% vs. 93.5%, Black: 16.8% vs. 4.3%; *p* < 0.001). IS patients had more smokers (12.1% vs. 3.2%; *p* = 0.001), fewer recurrent hernias (45.0% vs. 69.7%; *p* < 0.001), and smaller defects (155.7 ± 142.2 vs. 256.4 ± 202.9 cm^2^; *p* < 0.001). Wound class, mesh type, and rate of fascial closure were similar, but IS patients underwent fewer panniculectomies (13.4% vs. 34.1%; *p* < 0.001), component separations (26.2% vs. 51.4%; *p* < 0.001), received smaller mesh (744.2 ± 495.6 vs. 975.7 ± 442.3 cm^2^; *p* < 0.001), and had shorter length-of-stay (4.8 ± 2.0 vs. 7.0 ± 5.5 days; *p* < 0.001). There was no difference in wound breakdown, seroma requiring intervention, hematoma, mesh infection, or recurrence; however, IS patients had decreased wound infections (2.0% vs. 8.6%; *p* = 0.009), overall wound complications (11.4% vs. 21.1%; *p* = 0.016), readmissions (2.7% vs. 13.0%; *p* = 0.001), and reoperations (3.4% vs. 11.4%; *p* = 0.007). Of MISR patients, 80.9% were IS and 19.1% were OOS. In contrast to OVHR, MISR IS and OOS patients had similar demographics, preoperative characteristics, intraoperative details, and postoperative outcomes.

**Conclusion:** Although there were no differences in referred patients for MISR, this study demonstrates the racial disparities that exist among our IS and OOS complex, open AWR patients. Awareness of these disparities can help clinicians work towards equitable access to care and equal referrals to tertiary hernia centers.

## Introduction

Health disparities permeate many facets of surgical care, thus social determinants of health have become a frequently discussed topic in hopes to improve health equity and access to care. These disparities predominantly revolve around racial and socioeconomic inequities, which have been particularly discussed in the setting of emergency general surgery [1–3].

Specifically, disparities have been frequently reported in incisional hernia management. It has been demonstrated that Black patients were more likely to present with acute incarceration requiring emergent repair and resulting in greater complications, while White patients were more likely to undergo elective repair [4–8]. Click or tap here to enter text. Our prior work employed the National Surgical Quality Improvement Program (NSQIP) database to evaluate the longevity and potential change of this racial disparity over time [9]. Consistent with other data [4–6], Black and Hispanic patients were more likely to require emergent ventral hernia repair compared to their White counterparts; a national trend which unfortunately showed no improvement from 2008 to 2019 [9].

Socioeconomic status (SES), which can be defined by a variety of methods, has also been associated with inequitable elective hernia repair [8]. One surrogate of SES was utilizing zone improvement plan (zip) codes to obtain estimated household income [4, 10]. Handzel et al. found that patients with higher income were more likely to undergo elective hernia repair [4]. Insurance status, another surrogate for SES, represented a modifiable risk factor in published literature and has been shown to impact hernia management and outcomes [11, 12]. Lack of insurance was associated with more than twice the rate of emergent repair as well as increased serious adverse effects [13]. Medicaid and Medicare were predictors of postoperative complications, such as reoperation, readmission, and emergency department visit, when compared to private payer status [5, 6, 11–14].

Equal access to minimally invasive surgery has also been a concern [1, 9, 15]. Several studies have demonstrated a racial disparity in the laparoscopic or robotic approach for common intraabdominal surgeries [1, 16, 17]. Tatebe et al. evaluated the socioeconomic factors influencing the management and outcomes of paraesophageal hernia repairs by comparing county and private hospitals [15]. Within each respective hospital, there were no disparities in access to robotic repair; however, overall factors associated with robotic surgery included private hospital location, increased income, and private insurance status [15]. Vu et al. noted similar findings, where Black patients were less likely to undergo minimally invasive inguinal hernia repair, as a result of disparate access to expert minimally invasive surgeons [17]. For ventral and incisional hernias nationally, laparoscopy was more commonly utilized in White patients compared to Black or Hispanic patients, though this incongruity appeared to be slowly improving based on Katzen et al.’s review of the NSQIP database [9, 16].

The aforementioned literature has documented several disparities that exist in emergency general surgery, specifically ventral hernia repair; but little evidence on disparities in referral patterns for elective abdominal wall reconstruction (AWR) has been reported. The goal of this study was to evaluate the potential racial and demographic disparities in patients referred to our own tertiary hernia center. To do so, we examined the characteristics and outcomes of our in-state and out-of-state referral populations. We hypothesized that our patients travelling from out-of-state for care were less likely be of a racial minority and were more likely to have private insurance, a surrogate for SES.

## Methods

### Patient Selection and Study Design

Institutional review board (IRB) approval was obtained prior to the beginning of this study. Patients provided written informed consent to participate in this study and have their information documented in our institutional database.

This study was conducted at a tertiary care hernia center in North Carolina, which is home to a multidisciplinary AWR program. The patient population at this facility is comorbid with complex hernias. Given the expertise of this AWR program, particularly in open preperitoneal ventral hernia repairs, patients from across the country are referred to this institution for hernia management.

A prospectively maintained institutional database was queried for patients who underwent open, laparoscopic, and robotic ventral hernia repair from 1 January 2011 to 31 December 2022. Patients were included if they had documented insurance status and address information. Patients with other types of hernias were excluded from the study. Patients were divided into in-state (IS) referrals and out-of-state (OOS) referrals based on their home address at the time of surgery and were compared. The distance from patients’ home zip codes to the hospital address zip code was calculated for every patient in the most direct path between the two points. Open ventral hernia repairs (OVHR) were evaluated separately from minimally invasive repairs (MISR), which included both laparoscopic (LVHR) and robotic ventral hernia repairs (RVHR).

The primary aim was to assess the demographic differences in our in-state referrals when compared to our out-of-state referrals, particularly race and socioeconomic status. Insurance payer was categorized into private and commercial insurance, Medicare, or public assistance, which included Medicaid, self-pay, Veterans Affairs insurance, and worker’s compensation. Operative characteristics and postoperative outcomes were also reviewed. Overall wound complications were defined as any incident of wound breakdown, infection, cellulitis, seroma or hematoma requiring intervention, or mesh infection. Data was reported as in-state versus out-of-state.

### Statistical Analysis

Standard statistical methods and descriptive statistics were used for this study. Between-group comparisons were performed and analyzed by a trained statistician using Statistical Analysis Software (SAS Version 9.4). Fisher’s exact tests and Chi-Square were applied to analyze categorical variables, which were reported as percentages. While Kruskal-Wallis were utilized to compare continuous variables and were reported as mean values with corresponding standard deviations. All *p*-values were two-sided. Statistical significance was set at *p* < 0.05.

## Results

**FIGURE 1:**
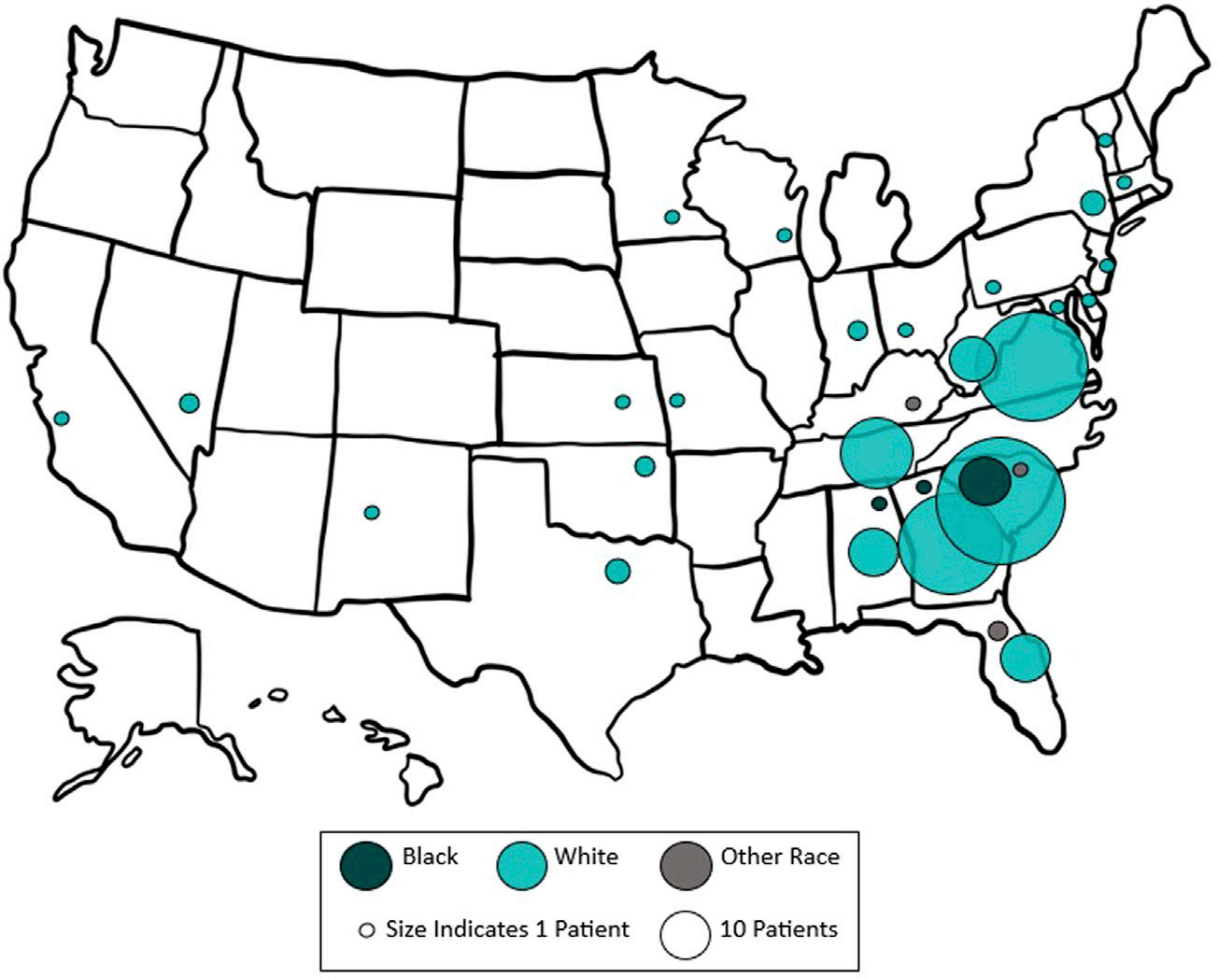
Map of out-of-state referrals to our tertiary hernia center by race. The approximate size of each circle represents the number of patients referred from each state.

A total of 554 patients met inclusion criteria. Of these, 334 underwent OVHR and 220 underwent MISR. The majority of all patients, 59.0% (*n* = 327), were IS, while 41.0% (*n* = 227) were from OOS. IS patients traveled a minimum of 2.0 km and maximum of 397.9 km to the hernia center. OOS patients traveled a minimum of 23.1 km and maximum of 3662.9 km (Figure 1). IS patients were more likely to undergo MISR (OVHR: 45.6% vs. 81.5%, LVHR: 38.2% vs. 14.1%, RVHR: 16.2% vs. 4.4%; *p* < 0.001) when compared to OOS referrals.

### Open Ventral Hernia Repairs

After review, 334 patients underwent OVHR; 44.6% (*n* = 149) were IS referrals and 55.4% (*n* = 185) were OOS referrals. Patients’ average age (56.6 ± 12.3 vs. 58.8 ± 11.4 years; *p* = 0.075), body mass index (BMI) (31.7 ± 7.0 vs. 32.8 ± 7.0 kg/m^2^; *p* = 0.168), and sex (53.0% vs. 50.3% female; *p* = 0.617) were similar between IS and OOS groups. IS patients were statistically more likely to be Black or of another racial minority compared to the OOS patients (White: 77.9% vs. 93.5%, Black: 16.8% vs. 4.3%, other race: 5.4% vs. 2.2%; *p* < 0.001). IS patients traveled shorter average distances to reach the hernia center (67.4 ± 75.5 vs. 451.6 ± 532.7 km; *p* < 0.001). Insurance payer was not statistically different between IS and OOS (private insurance: 55.0% vs. 51.4%, Medicare: 36.9% vs. 44.9%, public assistance: 8.1% vs. 3.8%; *p* = 0.119). IS patients were more often current smokers (12.1% vs. 3.2%; *p* = 0.001), but there was no difference in rate of diabetes (28.2% vs. 21.1%; *p* = 0.158) or American Society of Anesthesiologists (ASA) scores (III: 54.4% vs. 51.4%; *p* = 0.541). IS patients had smaller defects (155.7 ± 142.2 vs. 256.4 ± 202.9 cm^2^; *p* < 0.001) and were less likely to have had a prior ventral hernia repair (45.0% vs. 69.7%; *p* < 0.001). There was no difference in use of preoperative abdominal wall Botulinum Toxin A injection (2.7% vs. 3.8%; *p* = 0.760).

Intraoperatively, there was no difference in Centers for Disease Control (CDC) wound class (clean: 81.9% vs. 81.1%; *p* = 0.893), mesh type (75.2% vs. 82.2% synthetic; *p* = 0.101), rate of fascial closure (98.0% vs. 97.3%; *p* = 0.736), or delayed primary closure (7.4% vs. 4.9%; *p* = 0.488), but IS patients had a lower rate of preperitoneal repair (preperitoneal: 97.3% vs. 100.0%, intraperitoneal: 2.7% vs. 0.0%; *p* = 0.039), panniculectomy (13.4% vs. 34.1%; *p* < 0.001), and component separation (26.2% vs. 51.4%; *p* < 0.001). IS also underwent shorter operations (147.8 ± 60.2 vs. 200.2 ± 86.9 min; *p* < 0.001), received smaller mesh (744.2 ± 495.6 vs. 975.7 ± 442.3 cm^2^; *p* < 0.001), and required shorter length-of-stay (4.8 ± 2.0 vs. 7.0 ± 5.5 days; *p* < 0.001).

There was no difference in postoperative rates of wound breakdown (2.7% vs. 7.0%; *p* = 0.082), cellulitis (2.0% vs. 3.8%; *p* = 0.521), seroma requiring intervention (5.4% vs. 9.2%; *p* = 0.177), hematoma (2.7% vs. 3.2%; *p* > 0.999), intraabdominal abscess (1.3% vs. 2.7%; *p* = 0.465), or mesh infection (0.0% vs. 2.7%; *p* = 0.067) between IS and OOS patients. However, IS patients had decreased wound infections (2.0% vs. 8.6%; *p* = 0.009) and overall wound complications (11.4% vs. 21.1%; *p* = 0.016). IS patients had fewer readmissions (2.7% vs. 13.0%; *p* = 0.001) and reoperations (3.4% vs. 11.4%; *p* = 0.007) but no difference in hernia recurrence (1.3% vs. 5.4%; *p* = 0.073). IS patients had longer follow-up (10.8 ± 14.2 vs. 8.8 ± 16.7 months; *p* = 0.011) (Table 1).

**TABLE 1:** Clinical information and outcomes of open ventral hernia repairs at our tertiary referral hernia center.

	Demographic information**		
	In-State (*n* = 149)	Out-of-State (*n* = 185)	*p-*value
Race			<0.001
White	116 (77.9%)	173 (93.5%)	
Black	25 (16.8%)	8 (4.3%)	
Other Races	8 (5.4%)	4 (2.2%)	
Distance Traveled			<0.001
Miles	41.9 ± 46.9	280.6 ± 331.0	
Kilometers	67.4 ± 75.5	451.6 ± 532.7	
Insurance Payer			0.119
Private	82 (55.0%)	95 (51.4%)	
Medicare	55 (36.9%)	83 (44.9%)	
Public Assistance^a^	12 (8.1%)	7 (3.8%)	
Smoking Status			0.001
Never Smoker	83 (55.7%)	128 (69.2%)	
Former Smoker	48 (32.2%)	40 (21.6%)	
Current Smoker	18 (12.1%)	6 (3.2%)	
ASA* Score			0.318
I	3 (2.0%)	1 (0.5%)	
II	60 (40.3%)	84 (45.4%)	
III	81 (54.4%)	95 (51.4%)	
IV	5 (3.4%)	5 (2.7%)	
Hernia Defect Size (cm^2^)	155.7 ± 142.2	256.4 ± 202.9	<0.001
Recurrent Hernia	67 (45.0%)	129 (69.7%)	<0.001
	Intraoperative details**		
Wound Class			0.893
Clean	122 (81.9%)	150 (81.1%)	
Clean-Contaminated	10 (6.7%)	16 (8.6%)	
Contaminated	8 (5.4%)	10 (5.4%)	
Dirty-Infected	9 (6.0%)	9 (4.9%)	
Mesh Type			0.101
Synthetic	112 (75.2%)	152 (82.2%)	
Biologic	37 (24.8%)	31 (16.8%)	
No Mesh	0 (0.0%)	2 (1.1%)	
Mesh Location			0.039
Preperitoneal	145 (97.3%)	185 (100.0%)	
Intraperitoneal	4 (2.7%)	0 (0.0%)	
Mesh Size (cm^2^)	744.2 ± 495.6	975.7 ± 442.3	<0.001
Panniculectomy	20 (13.4%)	63 (34.1%)	<0.001
Component Separation	39 (26.2%)	95 (51.4%)	<0.001
Operative Time (minutes)	147.8 ± 60.2	200.2 ± 86.9	<0.001
	Postoperative outcomes**		
Length-of-Stay (days)	4.8 ± 2.0	7.0 ± 5.5	<0.001
Wound Complications^b^	17 (11.4%)	39 (21.1%)	0.016
Wound Infection	3 (2.0%)	16 (8.6%)	0.009
Readmission	4 (2.7%)	24 (13.0%)	0.001
Reoperation	5 (3.4%)	21 (11.4%)	0.007
Recurrence	2 (1.3%)	10 (5.4%)	0.073
Follow-Up (months)	10.8 ± 14.2	8.8 ± 16.7	0.011

*OVHR, open ventral hernia repair; ASA, American society of anesthesiologists.

**Data are presented as n(%) or mean ± SD.

^a^ Compilation of self-pay, workers’ compensation, Medicaid, and Veterans Affairs coverage.

^b^ Compilation of wound breakdown, cellulitis, wound infection, seroma requiring intervention, hematoma, mesh infection.

### Minimally Invasive Ventral Hernia Repairs

A total of 220 patients underwent MISR, with 71.4% (*n* = 157) undergoing LVHR and 28.6% (*n* = 63) undergoing RVHR. Most MISR were performed on IS patients, 80.9% (*n* = 178), compared to 19.1% (*n* = 42) of OOS patients. In contrast to the findings in OVHR IS and OOS patients, there were no statistical differences in race between MISR IS and OOS patients (White: 70.2% vs. 83.3%, Black: 25.8% vs. 14.3%, other race: 3.9% vs. 2.4%; *p* = 0.225). Average age (57.2 ± 13.0 vs. 59.1 ± 12.9; *p* = 0.385), BMI (33.0 ± 7.4 vs. 38.4 ± 44.2 kg/m^2^; *p* = 0.298), sex (56.7% vs. 59.5% female; *p* = 0.743), and insurance payer (private insurance: 52.2% vs. 40.5%, Medicare: 37.6% vs. 52.4%, public assistance: 10.1% vs. 7.1%; *p* = 0.362) were similar between IS and OOS. However, IS patients traveled statistically shorter distances to the hernia center (36.3 ± 39.2 vs. 124.8 ± 216.3 km; *p* < 0.001). There were no differences in rates of smoking (6.2% vs. 11.9%; *p* = 0.329), diabetes (23.6% vs. 16.7%; *p* = 0.332), ASA scores (III: 47.2% vs. 42.9%; *p* = 0.318), recurrent hernias (19.7% vs. 14.3%; *p* = 0.421), or defect size (29.3 ± 31.8 vs. 34.6 ± 33.9 cm^2^; *p* = 0.313).

Intraoperatively, there was no difference in CDC wound class (clean: 88.8% vs. 92.9%; *p* = 0.617), mesh type (91.6% vs. 88.1% synthetic; *p* = 0.086), mesh location (intraperitoneal: 68.5% vs. 60.5%; *p* = 0.227), mesh size (374.0 ± 225.4 vs. 352.4 ± 234.3 cm^2^; *p* = 0.566), rate of fascial closure (79.2% vs. 78.6%; *p* = 0.927), or component separation (3.9% vs. 2.4%; *p* > 0.999). Operative time (128.2 ± 71.8 vs. 135.4 ± 56.9 min; *p* = 0.203) and hospital length-of-stay (3.4 ± 2.5 vs. 3.3 ± 2.0 days; *p* = 0.904) were comparable between MISR IS and OOS patients.

Again, in contrast to the findings in the OVHR patients, there were no differences in rates of postoperative complications between IS and OOS patients. Specifically, wound breakdown (0.6% vs. 0.0%; *p* > 0.999), wound infection (1.7% vs. 0.0%; *p* > 0.999), cellulitis (10.7% vs. 0.0%; *p* > 0.999), seroma requiring intervention (0.6% vs. 0.0%; *p* > 0.999), hematoma (1.7% vs. 2.4%; *p* > 0.999), intraabdominal abscess (0.0% vs. 0.0%; *p* > 0.999), mesh infection (0.0% vs. 0.0%; *p* > 0.999), and overall wound complications (14.6% vs. 2.4%; *p* > 0.999) were comparable. MISR IS and OOS patients also had similar rates of readmissions (5.6% vs. 7.1%; *p* = 0.309), reoperations (1.1% vs. 0.0%; *p* > 0.999), hernia recurrence (7.3% vs. 0.0%; *p* > 0.999), and length of follow-up (41.4 ± 31.4 vs. 39.9 ± 36.5 months; *p* = 0.542) (Table 2).

**TABLE 2:** Clinical information and outcomes of minimally invasive ventral hernia repairs at our tertiary referral hernia center.

	Demographic information**		
	In-State (*n* = 178)	Out-of-State (*n* = 42)	*p-*value
Race			0.225
White	125 (70.2%)	35 (83.3%)	
Black	45 (25.8%)	6 (14.3%)	
Other Races	7 (3.9%)	1 (2.4%)	
Distance Traveled			<0.001
Miles	22.6 ± 24.4	77.6 ± 134.5	
Kilometers	36.3 ± 39.2	124.8 ± 216.3	
Insurance Payer			0.362
Private	93 (52.2%)	17 (40.5%)	
Medicare	67 (37.6%)	22 (52.4%)	
Public Assistance^a^	18 (10.1%)	3 (7.1%)	
Smoking Status			0.329
Never Smoker	116 (65.2%)	24 (57.1%)	
Former Smoker	51 (28.7%)	13 (31.0%)	
Current Smoker	11 (6.2%)	5 (11.9%)	
ASA* Score			0.318
I	10 (5.6%)	0 (0.0%)	
II	76 (42.7%)	23 (54.8%)	
III	84 (47.2%)	18 (42.9%)	
IV	8 (4.5%)	1 (2.4%)	
	Intraoperative details**		
Operative Technique			0.442
Laparoscopic	125 (70.2%)	32 (76.2%)	
Robotic	53 (29.8%)	10 (23.8%)	
Wound Class			
Clean	158 (88.8%)	39 (92.9%)	0.617
Clean-Contaminated	17 (9.6%)	2 (4.8%)	
Contaminated	3 (1.7%)	1 (2.4%)	
Dirty-Infected	0 (0.0%)	0 (0.0%)	
Mesh Type			0.086
Synthetic	163 (91.6%)	37 (88.1%)	
Biologic	2 (1.1%)	1 (2.4%)	
No Mesh	13 (7.3%)	4 (9.5%)	
Mesh Location			0.227
Intraperitoneal	115 (68.5%)	23 (60.5%)	
Preperitoneal	50 (29.8%)	14 (36.8%)	
Retrorectus	3 (1.8%)	0 (0.0%)	
Onlay	0 (0.0%)	1 (2.6%)	
	Postoperative outcomes**		
Length-of-Stay (days)	3.4 ± 2.5	3.3 ± 2.0	0.904
Wound Complications^b^	26 (14.6%)	1 (2.4%)	>0.999
Readmission	10 (5.6%)	3 (7.1%)	0.309
Reoperation	2 (1.1%)	0 (0.0%)	>0.999
Recurrence	13 (7.3%)	0 (0.0%)	>0.999
Follow-Up (months)	41.4 ± 31.4	39.9 ± 36.5	0.542

*MISR, minimally invasive surgical repair; ASA, American society of anesthesiologists.

**Data are presented as n(%) or mean ± SD.

^a^ Compilation of self-pay, workers’ compensation, Medicaid, and Veterans Affairs coverage.

^b^ Compilation of wound breakdown, cellulitis, wound infection, seroma requiring intervention, hematoma, mesh infection.

## Discussion

There are known racial and socioeconomic disparities in emergency general surgery, ventral hernia repair being no exception, but the goal of this study was to evaluate potential disparities among IS and OOS patients referred to a tertiary hernia center for elective, complex AWR. Ventral hernias can lead to significant financial burden and poor quality of life, thus the establishment of tertiary, regional referral centers has been particularly beneficial for patients with complex and burdensome defects [18, 19]. The multidisciplinary approach at such Centers of Excellence has contributed to improved patient outcomes, but what remains unclear is how equitable access to these centers really is [20, 21]. Although Shulkin et al. suggested that hernia centers are evenly distributed across the country, this initial investigation of our own hernia center suggests there remain disparities in availability to high-risk populations [19].

Ultimately, we found that in open AWR, there was a racial disparity between local patients and out-of-state patients; however, we did not find evidence of socioeconomic disadvantages, which we had hypothesized. IS patients had less complex, smaller hernias and ultimately underwent more MISR. The OOS patients were more likely to be White and had more complex hernias, as exemplified by their higher frequency of recurrent hernias, component separations, panniculectomies, larger defect sizes, and longer hospital length-of-stay. It was not unexpected then that these patients had increased wound complications, readmissions, and reoperations. OOS patients underwent more preperitoneal repairs, which we suspect was a result of OOS patients having larger hernias and being referred specifically for our expertise in this technique.

We had predicted that patients with private insurance or higher SES would be able to afford the time and cost to travel further to a specialty hernia center. However, there was no statistical difference between the IS and OOS insurance payer for OVHR. One potential contributor to this finding could be the impact of Medicare on SES. When older patients reach the age to qualify for Medicare, the disparities between private and public assistance coverage may be mitigated. As a hernia practice with older patients (average age was 57.7 ± 12.3 years for this entire cohort), insurance status may not represent an accurate surrogate of SES.

Additionally, we found that IS patients had greater length of follow-up, likely explained by the increased ease of local patients traveling to clinic. Whereas OOS patients require more time and effort to attend appointments and may choose to be evaluated closer to home. This finding, though, is different from our previous work, where more complex hernias with greater complications required more frequent visits and resulted in longer follow-up.

Although we saw a racial disparity in our OVHR IS and OOS referral populations, there was no evidence of disparate treatment by race or an overt explanation for this discrepancy. The etiology for racial and socioeconomic disparities remains multifactorial, including patient-factors, provider-biases, and systemic-level issues [9, 22, 23]. In an elective AWR practice, preoperative optimization is important for successful fascial closure and durable repair, but patients requiring optimization and their success may be influenced by race and SES [9, 24–27]. Preoperative optimization usually includes weight loss, smoking cessation, and glucose control. Yet, it has been demonstrated that racial minority patients and those from lower socioeconomic backgrounds have higher prevalence of obesity, tobacco use, and diabetes, putting them at increased odds of requiring optimization prior to hernia management [28–30]. Al-Mansour et al. sought to assess whether these at-risk populations were then less successful at achieving preoperative optimization goals [31]. Black race, female sex, and socioeconomic distress were factors associated with failure to meet at least one preoperative goal [31]. These findings reinforced the importance of a multidisciplinary practice to facilitate optimization in disadvantaged patients, who otherwise may have difficulty achieving eligibility for elective surgery [21, 32, 33].

Travel burden is another social determinant of health that may inhibit equitable access to hernia care. This was evidenced by the distance patients were required to travel for care, which may be unique to the United States as compared to Europe, where traveling is more feasible and accepted. Lussiez et al. evaluated surgical outcomes of patients in health professional shortage areas [34]. Expectedly, patients in health shortage areas traveled three times as far and twice as long for surgical care when compared to patients in more advantaged communities [34]. Although there were no differences in surgical outcomes, increased travel could discourage or prevent patients from accessing a tertiary hernia center. Patients from healthcare sparse areas have greater difficulty accessing primary care physicians, which may decrease the incidence in which they are referred for hernia care. Lack of personal transportation or unpredictability of public transportation can again impede OOS patients from accessing our tertiary hernia center. Data to further elucidate this disparity would be difficult to obtain, but it certainly is an important barrier to care that surgeons should consider.

We found evidence of a racial inequity in the referral patterns to our hernia center. There is no control at the surgeon-level regarding who was referred to us, suggesting that the origin of this problem occurs prior to surgical consultation. So then, why are Black patients not getting referred to OOS hernia centers like their White counterparts? There is little to no published literature on how or why patients are sent to local general surgeons as opposed to regional abdominal wall reconstruction-trained surgeons, and there remains no established protocol to guide general practitioners’ referrals. The referral system remains largely non-transparent, and it is difficult to know details such as how patients learned of our center, how many physicians they saw prior to us, or how long it took to be referred. Data in orthopedic surgery and bariatric surgery suggested that barriers to referral included lack of provider familiarity, minimal communication with subspecialists, and provider concerns or negative perceptions [35–38]. Further, there is no algorithm that primary care physicians can follow to best decide when to refer a patient out for management. Previous research in emergency medicine and cardiology has shown that healthcare providers’ implicit bias negatively altered their treatment of racial minorities and patients from low socioeconomic backgrounds [23, 39–41]. There is potential for bias in referral patterns for hernia repair too. Contrastingly, many patients do not have a primary care physician and instead self-refer to a surgeon via an internet search [42]. This introduces another realm of disparity in health literacy, which could impact where patients choose to go for their hernia care. Regardless of the etiology of this difference in OOS referral pattern, further research needs to be done to understand and hopefully dampen the disparity.

In the MISR patients, we did not find any statistical differences or disparities among the IS and OOS patients. This finding was surprising given the aforementioned literature that suggested there are racial and socioeconomic inequities in laparoscopic and robotic abdominal surgery [1, 9, 15–17]. Our tertiary referral center has expertise in open, complex AWR, which may explain our findings that more OOS patients were referred for OVHR rather than MISR. LVHR and RVHR is usually reserved for less complex hernias, which could suggest that OOS patients were appropriately managed by local surgeons and did not need referral to our center.

The limitations of this study include the retrospective nature and use of a single institution’s experience. These results may not be generalizable to other hernia Centers of Excellence or to other geographical locations, particularly outside of the United States. It would be interesting for other tertiary hernia centers to evaluate their referral demographics. Further, our institutional database only captures patients who undergo operative repair, thus we cannot make conclusions about the disparities among patients who are not offered surgery. Additionally, we included patients from South Carolina in the OOS group, although there are scenarios where those OOS patients were actually closer by distance to our hernia center than some patients from within North Carolina. Another limitation is the simplification of demographic data. For example, this study defined patients as White, Black, or other racial minority, but it does not consider multiracial patients; nor does this study have a comprehensive definition of SES. We utilized insurance payer as a surrogate, but there are several factors that contribute to SES. We initially discussed utilizing zip code to obtain average household income, but zip code alone may misrepresent SES. Future assessment could explore more accurate methods of measuring socioeconomic disadvantage. Collection of granular datapoints, such as transportation access, employment status, and health literacy, as well as more information on the referral process may also improve our interpretation of patients’ health disparities [6].

The goal of this study was to identify potential racial and socioeconomic disparities among in-state versus out-of-state referrals to a tertiary hernia center. However, the importance of this study is now to utilize this data to reduce barriers to care and improve equity in hernia management.

## Conclusion

Disparities continue to exist in elective abdominal wall reconstruction, as demonstrated by the racial disparity among our in-state and out-of-state open ventral hernia repair patients. This study raises awareness about the inherent possible biases and nonsystematic nature of the referral system. As a society, guardrails for seeking specialty referrals should be removed so clinicians can offer appropriate treatment in a timely manner. Identification of these disparities helps clinicians work towards equitable access to care, equal referrals to tertiary hernia centers, and ultimately improved hernia management.

## Data Availability

The raw data supporting the conclusion of this article will be made available by the authors, without undue reservation.
